# 3-(2-Aminophenyl)-4-methyl-1,3-thiazole-2(3H)-thione as an Ecofriendly Sulphur Transfer Agent to Prepare Alkanethiols in High Yield and High Purity

**DOI:** 10.3390/molecules14114634

**Published:** 2009-11-12

**Authors:** Mohammed Amine Mehdid, Ayada Djafri, Christian Roussel, Federico Andreoli

**Affiliations:** 1 Laboratoire de synthèse organique appliquée, Département de Chimie, Faculté de Sciences, Université d’Oran-es-Senia, Algeria; E-Mails: mmehdid@yahoo.fr (M.A.M.); djafriayada@yahoo.fr (A.D); 2 ISM2, Chirosciences, Université Paul Cézanne Aix-Marseille III, 13397 Marseille CEDEX 20, France; E-Mail: christian.roussel@univ-cezanne.fr (C.R.)

**Keywords:** thiols, dithiols, sulphur transfer, thiazoline-2-thione

## Abstract

A new process is described for preparing very pure linear alkanethiols and linear α,ω-alkanedithiols using a sequential alkylation of the title compound, followed by a ring closure to quantitatively give the corresponding 3-methyl[1,3]thiazolo[3,2-a]-[3,1]benzimidazol-9-ium salt and the alkanethiol derivative under mild conditions. The alkanethiol and the heteroaromatic salt are easily separated by a simple extraction process. The intermediate thiazolium quaternary salts resulting from the first reaction step can be isolated in quantitative yields, affording an odourless protected form of the thiols.

## Introduction

Long-chain *n*-alkanethiols and α,ω-alkanedithiols are valuable compounds in material sciences due to their ability to bind on gold surfaces [1,2,3,4,5,6,7,8]. In relation with the demand for very pure *n*-alkanethiols and *α,ω*-alkanedithiols, new methods for the synthesis of these molecules have been developed, furnishing good alternatives to classical ones [9,10,11]. Among them, the trimethylsilylthioxy-dehalogenation reaction reported by Hu and Fox [12], the direct synthesis from alcohols by Bandgar, Sadavarte and Uppalla [13], and the SmI_2_-promoted reductions of sodium alkyl thiosulfates and alkyl thiocyanates of Zhan, Lang, Liu and Hu [14], give alkanethiols in average to good yields; however, for all these methods, distillation or column chromatography is needed to further purify the final product. The one pot conversion of alkyl halides into thiols proposed by Molina, Alajarin and Vilaplana [15] doesn’t require final purification steps, but suffers from low yields (lower than 66%) when applied to the synthesis of long chain *n*-alkanethiols.

Moreover, it must be stressed that thiols easily undergo oxidation to disulphides under ambient conditions. Protecting groups are thus used to grant long term stability to derivatives, implying development of protection-deprotection procedures [16,17,18,19,20] with consequent loss of yield.

Herein we report a new *n*-alkanethiol and linear *α,ω*-alkanedithiol synthesis which involves the formation of a (bis-)thiazolium salt, starting from 3-(2-aminophenyl)-4-methyl-1,3-thiazole-2(3*H*)-thione (**1**) and the corresponding (di)halide. The reactions are carried out under mild conditions and no inert conditions are needed; the final products are recovered in their pure form by simple extraction.

## Results and Discussion

**Scheme 1:**
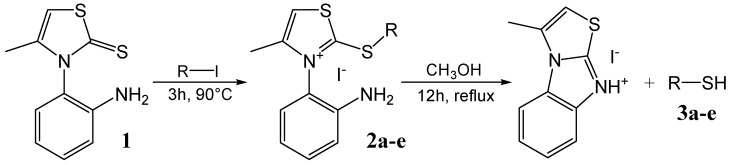
*n*-Alkanethiols synthesis via thiazolium iodides.

3-(2-Aminophenyl)-4-methyl-1,3-thiazole-2(3*H*)-thione (**1**) represents a cornerstone for different projects in progress in our laboratory [21,22,23,24,25]. Firstly described by Bellec *et al*. [26], the synthetic route to **1** starts from readily available material (CS_2_, chloracetone and phenylene-1,2-diamine); this synthesis has been further optimised, leading to crystallised thiazoline-2-thione **1** in 75% isolated yield. We have described that the reaction of **1** with methyliodide gave a salt **2** (R = Me, Scheme 1) which was cleanly transformed into thiazolobenzimidazole affording a new and efficient access to that aromatic tricyclic framework [22]. The only by-product was methanethiol, which escaped from the reaction medium and was not characterized. If one considers the two step process described in Scheme 1, the thiazoline-2-thione **1** is thus acting as a sulphur transfer agent which mediates the transformation of an alkyl halide into an alkanethiol. The sulphur transfer is occurring through salt **2**, which can be considered as a protected form of the alkanethiol. We thus wondered if this procedure might lead to a general and clean synthesis of long-chain *n*-alkanethiols.

A series of different linear alkyliodides was reacted with thiazolinethione **1** to produce the derived thiazolium iodides **2** in high yield. In a second step, under heating in refluxing methanol, the thiazolium iodides **2** were quantitatively transformed in a known thiazolobenzimidazolium salt and the corresponding thiols (Scheme 1).

The results obtained are summarized in Table 1.

In the first step, the solvent free reaction of thiazoline-2-thione **1** with an excess of the corresponding alkyliodide (Scheme 1) generates the respective thiazolium iodides **2**. TLC monitoring confirmed that, for all entries, the starting material **1** completely disappeared after 3 h at 90 °C. Salts **2** were then isolated in very good yields (Table 1) by simple filtration over silica gel and fully characterised. The purification step allows one to recover and to reuse all the excess of alkyliodide, so that the molar ratio **1**/(alkylating agent) can be brought closer to 1/1. Moreover, removal of the excess halide from the medium prevents the formation of unwanted dialkylsulphides, once the thiol is formed in the second step. Last but not least, iodides **2** are stable and odourless compounds that can be stored at 3-4 °C without any risk of alteration, thus the thiazoline moiety acts as a protecting group for the thiol.

Thiazolium iodides **2** are then easily converted by cyclization in methanol under reflux into the thiazolobenzimidazolium iodide in 12 h, releasing the corresponding thiol (second step, Scheme 1). Thiols **3a-e** were isolated as pure compounds in yields higher than 90% (Table 1) by simple extraction with Et_2_O. No formation of disulphide by-product was observed in the final products. However when the reaction was performed using various substituted benzylchlorides as alkylating agent for **1** a mixture of substituted benzylmercaptans and substituted dibenzyldisulphides was obtained (data not shown).

**Scheme 2:**
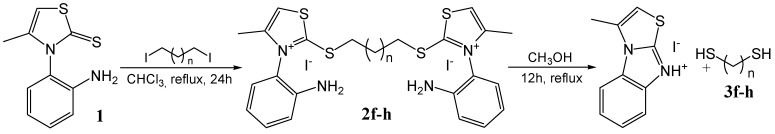
Dithiols synthesis via bis-thiazolium diiodides.

The good results obtained for *n*-alkanethiols prompted us to apply this method to the synthesis of *α,ω*-alkanedithiols. In this case, the reaction conditions were changed, due to the need to obtain bis-thiazolium salts. Thiazoline-2-thione **1** was then reacted with 1/2 mole of *α,ω*-alkyldiiodides in chloroform under reflux (Scheme 2).

Once again, the complete disappearance of the starting product was monitored by TLC. Two different work ups were successively used to isolate the obtained products (see Experimental section). ^1^H-NMR spectra were recorded for each compound **2f**-**h**, confirming the presence of the corresponding bis-thiazolium diiodides as unique compounds. The salts **2f**-**h** were then fully characterised and the respective yields are reported in Table 2. It should be noted that bis-thiazolium diiodides **2** might be obtained as a mixture of diastereomers (meso and *d*, *l*) since the starting thiazoline-2-thione **1** is chiral [10]. A detailed analysis of this mixture was beyond the scope of this paper since the cyclization step leading to *α,ω*-alkanedithiols yielded the same achiral compounds starting from either diastereomer.

Like thiazolium salts **2a-e**, bis-thiazolium salts **2f-h** can be stored at 3-4°C without any risk of alteration and show all the advantages mentioned for **2a-e**. The same procedure and work up used to obtain thiols is then applied to the synthesis of *α,ω*-alkanedithiols: after 2h under reflux in MeOH, the cyclization to thiazolobenzimidazole is complete and *α,ω*-alkanedithiols **3f**-**3h** are recovered by simple extraction with Et_2_O. It must be stressed that also in this case no formation of by-products derived from sulphur oxidation was observed.

## Experimental 

### General

^1^H-NMR spectra were recorded at 500, 300 or 200 MHz and ^13^C-NMR spectra at 125, 75 or 50 MHz on Bruker Avance DRX-500, DPX-300 or 200 instruments, respectively. Chemical shifts are reported in ppm with the signal for residual solvent as internal standard. *J* values are reported in Hz. High resolution mass spectra were performed on Q-STAR Elite spectrometer. Melting points were measured using a Büchi Melting Point B-545 apparatus. Filtrations through silica gel were performed with silica gel 60 (230-400 mesh). TLCs were carried out on Merck 60F254 silica plates. 3-(2-Aminophenyl)-4-methyl-1,3-thiazole-2(3H)-thione (**1**) was prepared according to reference [22]. All the halogen compounds were commercially available (Sigma-Aldrich, Alfa Aesar).

### General procedure for the synthesis of monothiazolium iodides ***2a**-**e***

3-(2-Aminophenyl)-4-methyl-1,3-thiazole-2(3*H*)-thione (**1**, 300 mg, 1.35 mmol) was suspended in the corresponding iodoalkane (for **2a**: *n*-heptyliodide, 2.4 mL, 14.64 mmol; for **2b**: *n*-nonyliodide, 2.8 mL, 14.17 mmol; for **2c**: *n*-decyliodide, 2.8 mL, 13.12 mmol; for **2d**: *n*-dodecyliodide, 2.8 mL, 11.35 mmol; for **2e**: *n*-octadecyliodide, 3.8 g, 9.99 mmol) and the mixture stirred at 90 °C. After 3 h, the mixture is cooled to r.t. and CH_2_Cl_2_ (3 mL) is added to completely dissolve the precipitate formed. The solution is filtered on silica gel to remove the excess of alkyl halide (using CH_2_Cl_2_). Then the silica is washed with MeOH and the corresponding thiazolium iodide is recovered after evaporation of the solvent.

*3-(2-Aminophenyl)-2-(heptylthio)-4-methylthiazol-3-ium iodide* (**2a**): Yield: 96% (581 mg, orange solid); mp: 53-55°C; ^1^H-NMR (300 MHz, CDCl_3_) δ = 0.85 (t, 3H, *J* = 6.7; CH_3_), 1.25-1.47 (m, 8H; 4(CH_2_)), 1.77-1.88 (m, 2H; CH_2_), 2.26-2.27 (d, 3H, *J* = 1.0; CH_3_), 3.27-3.59 (m, 2H; SCH_2_), 4.79 (s, 2H; NH_2_), 6.79-6.84 ( m, 1H; Ar), 6.93-7.00 (m, 2H; Ar), 7.33-7.38 (m, 1H; Ar), 7.92 (q, 1H, *J* = 1.0; =CH); ^13^C-NMR (75 MHz, CDCl_3_) δ = 14.12, 14.37, 22.59, 27.62, 28.75, 28.83, 31.47, 36.82, 118.37, 118.53, 118.64, 118.83, 126.87, 133.19, 143.16, 145.67, 177.91; HRMS m/z calcd C_17_H_25_N_2_S_2_^+^ [M-I]^+^: 321.1454, found: 321.1455.

*3-(2-Aminophenyl-2-(nonylthio)-4-methylthiazol-3-ium iodide* (**2b**): Yield: 96% (617 mg, orange solid); mp: 51-53°C; ^1^H-NMR (300 MHz, CDCl_3_) δ = 0.85 (t, 3H, *J* = 6.7; CH_3_), 1.23-1.45 (m, 12H; 6(CH_2_)), 1.76-1.87 (m, 2H; CH_2_), 2.26 (d, 3H, *J* = 0.9; CH_3_), 3.26-4.11 (m, 4H; SCH_2_+NH_2_), 6.78-6.84 ( m, 1H; Ar), 6.93-7.00 ( m, 2H; Ar), 7.32-7.38 ( m, 1H; Ar), 7.93 (q, 1H, *J* = 0.9; =CH); ^13^C-NMR (75 MHz, CDCl_3_) δ = 14.18, 14.35, 22.71, 27.62, 28.87, 29.09, 29.23, 29.35, 31.87, 36.81, 118.37, 118.52, 118.65, 118.88, 126.86, 133.17, 143.16, 145.65, 177.88; HRMS m/z calcd C_19_H_29_N_2_S_2_^+^ [M-I]^+^: 349.1767, found: 349.1767.

*3-(2-Aminophenyl)-2-(decylthio)-4-methylthiazol-3-ium iodide* (**2c**): Yield: 94% (622 mg, orange solid); mp: 52-54 °C; ^1^H-NMR (300 MHz, CDCl_3_) δ = 0.85 (t, 3H, *J* = 6.6; CH_3_), 1.22-1.44 (m, 14H; 7(CH_2_)), 1.76-1.86 (m, 2H; CH_2_), 2.26 (d, 3H, *J* = 0.7; CH_3_ ), 3.26-3.58 (m, 2H; SCH_2_), 4.79 (s, 2H; NH_2_), 6.78-6.83 (m, 1H; Ar), 6.93-7.01 (m, 2H; Ar), 7.32-7.37 (m, 1H; Ar), 7.95 (q, 1H, *J* = 0.7; =CH); ^13^C-NMR (75 MHz, CDCl_3_) δ = 14.17, 14.34, 22.71, 27.60, 28.86, 29.07, 29.31, 29.37, 29.50, 31.90, 36.79, 118.35, 118.50, 118.63, 118.97, 129.85, 133.14, 143.15, 145.59, 177.81; HRMS m/z calcd C_20_H_31_N_2_S_2_^+^ [M-I]^+^: 363.1923, found: 363.1922.

*3-(2-Aminophenyl)-2-(dodecylthio)-4-methylthiazol-3-ium iodide* (**2d**): Yield: 93% (651 mg, orange solid); mp: 50-52°C; ^1^H-NMR (300 MHz, CDCl_3_) δ = 0.86 (t, 3H, *J* = 6.7; CH_3_), 1.23-1.45 (m, 18H; 9(CH_2_)), 1.77-1.88 (m, 2H; CH_2_), 2.27 (d, 3H, *J* = 0.7; CH_3_), 3.27-3.60 (m, 2H; SCH_2_), 4.81 (s, 2H; NH_2_), 6.79-6.84 (m, 1H; Ar), 6.93-6.99 (m, 2H; Ar), 7.33-7.39 ( m, 1H; Ar), 7.90 (q, 1H, *J* = 0.7; =CH); ^13^C-NMR (75 MHz, CDCl_3_) δ = 14.22, 14.36, 22.77, 27.64, 28.90, 29.11, 29.41 (2C), 29.59, 29.69 (2C), 31.99, 36.84, 118.37, 118.50, 118.64, 118.81, 126.87, 133.18, 143.20, 145.68, 177.96; HRMS m/z calcd C_22_H_35_N_2_S_2_^+^ [M-I]^+^: 391.2236, found: 391.2237.

*3-(2-Aminophenyl-2-(octadecylthio)-4-methylthiazol-3-ium iodide* (**2e**): Yield: 98% (797 mg, orange solid); mp: 51-53 °C; ^1^H-NMR (300 MHz, CDCl_3_) δ = 0.86 (t, 3H, *J* = 6.7; CH_3_), 1.18-1.46 (m, 30H; 15(CH_2_)), 1.46-1.78 (m, 2H; CH_2_), 2.27-2.28 (d, 3H, *J* = 0.9; CH_3_), 3.27-3.61 (m, 2H; SCH_2_), 4.78 (s, 2H; NH_2_), 6.79-6.85 (m, 1H; Ar), 6.93-6.99 (m, 2H; Ar), 7.34-7.39 (m, 1H; Ar), 7.88 (q, 1H, *J* = 0.9; =CH); ^13^C-NMR (75 MHz, CDCl_3_) δ = 14.24, 14.40, 22.80, 27.65, 28.92, 29.13, 29.44, 29.48, 29.62, 29.73, 29.77 (brs), 29.81 (brs), 32.04, 36.88, 118.36, 118.54, 118.61 (2C), 126.87, 133.23, 143.17, 145.77, 178.05; HRMS m/z calcd C_28_H_47_N_2_S_2_^+^ [M-I]^+^: 475.3175, found: 475.3166.

### Procedure for the synthesis of bis-thiazolium diiodides ***2f** and **2g***

3-(2-Aminophenyl)-4-methyl-1,3-thiazole-2(3*H*)-thione (**1**, 200 mg, 89.96 mmol) was dissolved in CHCl_3_ (5 mL), then the corresponding α,ω-diiodide is added (for **2f**: 1,3-diiodopropane, 52 μL, 0.5 eq; for **2g**: 1,4-diiodobutane, 59 μL, 0.5 eq) and the solution refluxed under magnetic stirring. After 24 h, the formed precipitate is filtered and washed with CHCl_3_ to yield the corresponding bis-thiazolium diiodide.

*2,2'-(Propane-1,3-diyldisulfanediyl)bis[3-(2-aminophenyl)-4-methyl-1,3-thiazol-3-ium] diiodide* (**2f**): Yield: 94% (313 mg, pale yellow solid); mp: 178-180°C (mixt.); ^1^H-NMR (500 MHz, CD_3_OD) δ = 2.25 (d, 6H, *J* = 0.9; 2CH_3_), 2.45-2.52 (m, 2H; CH_2_), 3.62 (t, 4H, *J* = 7.2; 2(SCH_2_)), 6.80-6.83 (m, 2H; Ar), 6.99-7.01 (m, 2H; Ar), 7.18-7.21 (m, 2H; Ar), 7.36-7.40 (m, 2H; Ar), 7.86 (q, 2H, *J* = 0.9; 2(=CH)); ^13^C-NMR (125 MHz, CDCl_3_) δ = 13.90 (2C), 27.31, 35.00 (2C), 118.55 (2C), 118.63 (2C), 118.79 (2C), 119.67 (2C), 128.53 (2C), 134.19 (2C), 145.26 (2C), 148.12 (2C), 179.08 (2C); HRMS m/z calcd C_23_H_26_N_4_S_4_^2+^ [M-2I]^2+^: 243.0515, found: 243.0519.

*2,2'-(Butane-1,4-diyldisulfanediyl)bis[3-(2-aminophenyl)-4-methyl-1,3-thiazol-3-ium] diiodide* (**2g**): Yield: 95% (322 mg, pale yellow solid); mp: 259-261 °C (mixt.); ^1^H-NMR (500 MHz, CD_3_OD) δ = 2.04-2.09 (m, 4H; 2(CH_2_)), 2.25 (d, 6H, *J* = 1.1; 2CH_3_), 3.47-3.54 (m, 4H; 2(SCH_2_)), 6.79-6.85 (m, 2H; Ar), 6.98-7.02 (m, 2H; Ar), 7.13-7.16 (m, 2H; Ar), 7.35-7.41 (m, 2H; Ar), 7.83 (q, 2H, *J* = 1.1; 2(=CH)); ^13^C-NMR (125 MHz, CDCl_3_) δ = 13.83 (2C), 27.74 (2C), 35.87 (2C), 118.10 (2C), 118.71 (2C), 118.87 (2C), 119.73 (2C), 128.39 (2C), 134.21 (2C), 145.27 (2C), 148.14 (2C), 179.61 (2C); HRMS m/z calcd C_24_H_28_N_4_S_4_^2+^ [M-2I]^2+^: 250.0593, found: 250.0586.

### Procedure for the synthesis of bis-thiazolium diiodide ***2h***

3-(2-Aminophenyl)-4-methyl-1,3-thiazole-2(3*H*)-thione (**1**, 200 mg, 89.96 mmol) was dissolved in CHCl_3_ (5 mL), then 1,5-diiodopentane (67 μL, 0.5 eq) is added and the solution refluxed under magnetic stirring. After 48 h, the solution is cooled to r.t. and CH_2_Cl_2_ (5 mL) is added. The solution is filtered on silica gel to remove the excess of reactive (using CH_2_Cl_2_). Then the silica is washed with MeOH and bis-thiazolium diiodide **2h** is recovered after evaporation of the solvent.

*2,2'-(Pentane-1,5-diyldisulfanediyl)bis[3-(2-aminophenyl)-4-methyl-1,3-thiazol-3-ium] diiodide* (**2h**): Yield: 89% (308 mg, orange solid); mp: 72-74°C (mixt.)C; ^1^H-NMR (200 MHz, CD_3_OD) δ = 1.58 (m, 2H; CH_2_), 1.89-2.03 (m, 4H; 2(CH_2_)), 2.24 (d, 6H, *J* = 1.0; 2CH_3_), 3.46 (t, 4H, *J* = 7.2; 2(SCH_2_)), 6.77-6.86 (m, 2H; Ar), 6.98-7.03 (m, 2H; Ar), 7.15-7.19 (m, 2H; Ar), 7.34-7.43 (m, 2H; Ar), 7.81 (q, 2H, *J* = 1.0; 2(=CH)); ^13^C-NMR (50 MHz, CD_3_OD) δ = 13.85 (2C), 28.20 (2C), 28.65, 36.47 (2C), 118.05 (2C), 118.69 (2C), 118.85 (2C), 119.78 (2C), 128.44 (2C), 134.14 (2C), 145.25 (2C), 147.97 (2C), 226.89 (2C); HRMS m/z calcd C_25_H_30_N_4_S_4_^2+^ [M-2I]^2+^: 257.0671, found: 257.0662.

### General procedure for the synthesis of n-alkanethiols

The corresponding thiazolium iodide (300 mg; **2a**: 0.67 mmol; **2b**: 0.63 mmol; **2c**: 0.61 mmol; **2d**: 0.58 mmol; **2e**: 0.50 mmol) was dissolved in MeOH (2.5 mL) and the solution refluxed under magnetic stirring. After 12 h, MeOH is evaporated and Et_2_O (20 mL) is added. The organic layer is washed with 5% HCl (3 × 15 mL) and brine (15 mL) and dried over MgSO_4_. Et_2_O is then evaporated to yield the corresponding thiols.

*1-Heptanethiol* (**3a**) [27]: Yield: 90% (80 mg, colourless oil); ^1^H-NMR (300 MHz, CDCl_3_) δ = 0.89 (t, 3H, *J* = 6.7; CH_3_), 1.26-1.43 (m, 9H; 4(CH_2_) + SH), 1.54-1.66 (m, 2H; CH_2_), 2.49-2.56 (m, 2H; SCH_2_); ^13^C-NMR (75 MHz, CDCl_3_) δ = 14.20, 22.75, 24.81, 28.50, 28.89, 31.88, 34.21.

*1-Nonanethiol* (**3b**) [28]: Yield: 92% (93 mg, colourless oil); ^1^H-NMR (300 MHz, CDCl_3_) δ = 0.88 (t, 3H, *J* = 6.6; CH_3_), 1.20-1.43 (m, 13H; 6(CH_2_) + SH), 1.55-1.65 (m, 2H; CH_2_), 2.48-2.55 (m, 2H; SCH_2_); ^13^C-NMR (75 MHz, CDCl_3_) δ = 14.24, 22.81, 24.80, 28.53, 29.23, 29.40, 29.62, 32.01, 34.21.

*1-Decanethiol* (**3c**) [27]: Yield: 94% (100 mg, colourless oil); ^1^H-NMR (300 MHz, CDCl_3_) δ = 0.87 (t, 3H, *J* = 6.6; CH_3_), 1.19-1.42 (m, 15H; 7(CH_2_) + SH), 1.55-1.65 (m, 2H; CH_2_), 2.48-2.55 (m, 2H; SCH_2_); ^13^C-NMR (75 MHz, CDCl_3_) δ = 14.23, 22.81, 24.79, 28.53, 29.22, 29.44, 29.66, 29.69, 32.03, 34.20.

*1-Dodecanethiol* (**3d**) [27]: Yield: 91% (106 mg, colourless oil); ^1^H-NMR (300 MHz, CDCl_3_) δ = 0.88 (t, 3H, *J* = 6.6; CH_3_), 1.19-1.43 (m, 19H; 9(CH_2_) + SH), 1.55-1.65 (m, 2H; CH_2_), 2.48-2.56 (m, 2H; SCH_2_); ^13^C-NMR (75 MHz, CDCl_3_) δ = 14.26, 22.83, 24.81, 28.54, 29.23, 29.49, 29.67, 29.74, 29.78, 29.79, 32.06, 34.21.

*1-Octadecanethiol* (**3e**) [28]: Yield: 92% (131 mg, white solid); mp: 30-32°C; ^1^H-NMR (300 MHz, CDCl_3_) δ = 0.88 (t, 3H *J* = 6.7; CH_3_), 1.19-1.43 (m, 31H; 15(CH_2_) + SH), 1.54-1.67 (m, 2H; CH_2_), 2.47-2.58 (m, 2H; SCH_2_); ^13^C-NMR (75 MHz, CDCl_3_) δ = 14.27, 22.85, 24.82, 28.54, 29.24, 29.52, 29.68, 29.75, 29.82 (3C), 29.85 (5C), 32.08, 34.22.

### General procedure for the synthesis of α,ω-alkanedithiols

The corresponding bis-thiazolium diiodide (300 mg; **2f**: 0.40 mmol; **2g**: 0.40 mmol; **2h**: 0.39 mmol) of are dissolved in MeOH (10 mL) and the solution refluxed under magnetic stirring. After 12 h, MeOH is evaporated and Et_2_O (20 mL) is added. The organic layer is washed with 5% HCl (3 × 20 mL) and brine (15 mL) and dried over MgSO_4_. Et_2_O is then evaporated to yield the corresponding dithiols.

*1,3-Propanedithiol* (**3f**) [28]: Yield: 91% (40 mg, colourless oil); ^1^H-NMR (300 MHz, CDCl_3_) δ = 1.33 (t, 2H, *J* = 8.0; 2(SH)), 1.86-1.95 (m, 2H; CH_2_), 2.63-2.70 (m, 4H; 2(SCH_2_)); ^13^C-NMR (75 MHz, CDCl_3_) δ = 23.01 (2C), 37.44.

*1,4-Butanedithiol* (**3g**) [27]: Yield: 90% (44 mg, colourless oil); ^1^H-NMR (300 MHz, CDCl_3_) δ = 1.35 (t, 2H, *J* = 7.8; 2(SH)), 1.70-1.76 (m, 4H; 2(CH_2_)), 2.51-2.58 (m, 4H; 2(SCH_2_)); ^13^C-NMR (75 MHz, CDCl_3_) δ = 24.24 (2C), 32.70 (2C).

*1,5-Pentanedithiol* (**3h**) [28]: Yield: 92% (49 mg, colourless oil); ^1^H-NMR (300 MHz, CDCl_3_) δ = 1.34 (t, 2H, *J* = 7.8; 2(SH)), 1.46-1.68 (m, 6H; 3(CH_2_)), 2.50-2.57 (m, 4H; 2(SCH_2_)); ^13^C-NMR (75 MHz, CDCl_3_) δ = 24.60 (2C), 27.18, 33.54 (2C).

## Conclusions

We have described a method that offers a new, mild, high yielding and particularly clean way to synthesize *n*-alkanethiols and linear *α,ω*-alkanedithiols through a sulphur transfer reaction. In addition, the possibility of isolating and storing the intermediate thiazolium salts offers a way to protect thiols in an odourless form.

## Figures and Tables

**Table 1:** Isolated yields in thiazolium iodides and thiols.

Reagent	Thiazolium salt	Yield (%)	Thiol	Yield (%)
CH_3_(CH_2_)_6_I	**2a**	96	**3a**	90
CH_3_(CH_2_)_8_I	**2b**	96	**3b**	92
CH_3_(CH_2_)_9_I	**2c**	94	**3c**	94
CH_3_(CH_2_)_11_I	**2d**	93	**3d**	91
CH_3_(CH_2_)_17_I	**2e**	98	**3e**	92

**Table 2:** Isolated yields of bis-thiazolium diiodides and α,ω-alkanedithiols.

Reagent	Thiazolium salt	Yield (%)	Thiol	Yield (%)
I(CH_2_)_3_I	**2f**	94	**3f**	91
I(CH_2_)_4_I	**2g**	95	**3g**	90
I(CH_2_)_5_I	**2h**	89	**3h**	92
